# *Salmonella* in Wild Boar Meat: Prevalence and Risk Assessment in Central Italy (Umbria and Marche Region)

**DOI:** 10.3390/foods13081156

**Published:** 2024-04-10

**Authors:** Caterina Altissimi, Sara Primavilla, Rossana Roila, Stefano Gavaudan, Benedetto Morandi, Stefania Di Lullo, Marta Coppini, Chiara Baldinelli, Dongjie Cai, Raffaella Branciari, Andrea Valiani, Peter Paulsen, David Ranucci

**Affiliations:** 1 Department of Veterinary Medicine, University of Perugia, Via San Costanzo 4, 06121 Perugia, Italy; caterina.altissimi@studenti.unipg.it (C.A.); marta.coppini@studenti.unipg.it (M.C.); raffaella.branciari@unipg.it (R.B.); david.ranucci@unipg.it (D.R.); 2 Istituto Zooprofilattico Sperimentale dell’Umbria e delle Marche “Togo Rosati”, 06121 Perugia, Italy; s.primavilla@izsum.it (S.P.); s.gavaudan@izsum.it (S.G.); b.morandi@izsum.it (B.M.); s.dilullo@izsum.it (S.D.L.); a.valiani@izsum.it (A.V.); 3 USLUmbria1—Igiene degli Alimenti di Origine Animale, Distretto Alto Chiascio, 06024 Gubbio, Italy; chiara.baldinelli@uslumbria1.it; 4 College of Veterinary Medicine, Sichuan Agricultural University, Chengdu 611130, China; dongjie_cai@sicau.edu.cn; 5 Unit of Food Hygiene and Technology, Centre for Food Science and Veterinary Public Health, Clinical Department for Farm Animals and Food System Science, University of Veterinary Medicine Vienna, 1210 Vienna, Austria; peter.paulsen@vetmeduni.ac.at

**Keywords:** game meat, food microbiology, risk analyses, foodborne pathogens

## Abstract

A survey was conducted from 2018 to 2023 to assess the presence of *Salmonella* in 280 hunted wild boar (carcasses after evisceration and skinning, N = 226; liver, N = 258; and fecal samples, N = 174). The overall prevalence was 2.86% (confidence interval 95%, 1.45–5.45%) with five positive samples detected in carcasses, three in the liver, and one in a fecal sample. This prevalence was in line with those found in nearby areas denoting a low number of positive samples. Positive animals were over 24 months of age and weighed, before skinning, 59.00 ± 9.11 Kg and no difference was detected in microbial loads between samples positive and negative for *Salmonella* (aerobic colony count of 4.59 and 4.66 log CFU/400 cm^2^, and *Enterobacteriaceae* count of 2.89 and 2.73 log CFU/400 cm^2^ (mean values) in positive and negative subjects, respectively). *Salmonella* Stanleyville was the most frequently isolated serotype. A semiquantitative risk assessment was conducted for the first time in game meat considering two products, meat cuts intended for cooking and fermented dry sausages. Only proper cooking can reduce the risk of ingestion of *Salmonella* to the minimum for consumers, whereas ready-to-eat dry sausages constitute risk products in terms of foodborne Salmonellosis (risk score of 64 out of 100).

## 1. Introduction

Wildlife acts as a reservoir for pathogens that could be spread to humans, domestic animals, and livestock. Specifically, wild boars seem to carry a variety of biological hazards [1,2], and, due to the significant increase in their abundance, it is crucial to identify and control risks to humans and the livestock sector. Furthermore, the exponential rise in wild boar populations necessitates tighter management and containment of this species, leading to greater availability of this meat [3]. Game meat is still, to date, a niche product primarily associated with regional culinary traditions, although modern consumers are becoming more interested in this kind of meat. This is due to its nutritional value, the ethicality of these animals not being raised intensively but free in their natural environment, and the lack of drugs (such as antibiotics) voluntarily administered to animals [4]. However, undercooked meats and typical game meat products, especially ready-to-eat raw cured meats, could carry foodborne pathogens [5].

Salmonellosis is an enteric infectious disease that poses a threat to public health. With 65,208 cases of human illness, 1014 foodborne outbreaks causing 6632 cases of illness, 1406 hospitalizations, and 8 deaths, it was the second-most-often reported foodborne zoonosis in the European Union in 2022; admittedly, game meat was not reported as a source [6]. Nonetheless, the possible increase in the amount of wild boar meat available in the market necessitates an accurate and continuous monitoring of *Salmonella* presence in the meat, with a risk-based approach to prevention and control of the hazard [7].

Several authors have investigated the prevalence of *Salmonella* in wild boars, with widely variable results depending on the sampled matrix, the geographic area investigated, and the proximity of farms and human settlements [2]. Feces are the most often investigated samples, although tonsils, spleen, kidney, and lymph nodes have also been considered [2]. Although Salmonella in animals is mainly present in the intestine with possible contamination of carcasses during hunting, handling, and evisceration procedures, it could be spread to other organs in live animals (particularly lymph nodes, but also the liver) [8].

Many authors have indirectly studied the prevalence of *Salmonella* spp. in wild boars, i.e., through serum samples [1,7,9]. However, these indicate the presence of antibodies against the microorganism but not the presence of *Salmonella* on carcass surfaces or meat. As reported in the literature, the prevalence of *Salmonella* on carcasses appears to be quite low, while highly variable results have been observed on meat [2,10,11].

This study retrospectively analyzed the prevalence of *Salmonella* spp. in wild boar over a 5-year period, in the areas between the Umbria and Marche regions (central Italy). A semi-quantitative risk assessment was proposed for the human population of these regions.

## 2. Materials and Methods

### 2.1. Samples Collection

**Figure 1:**
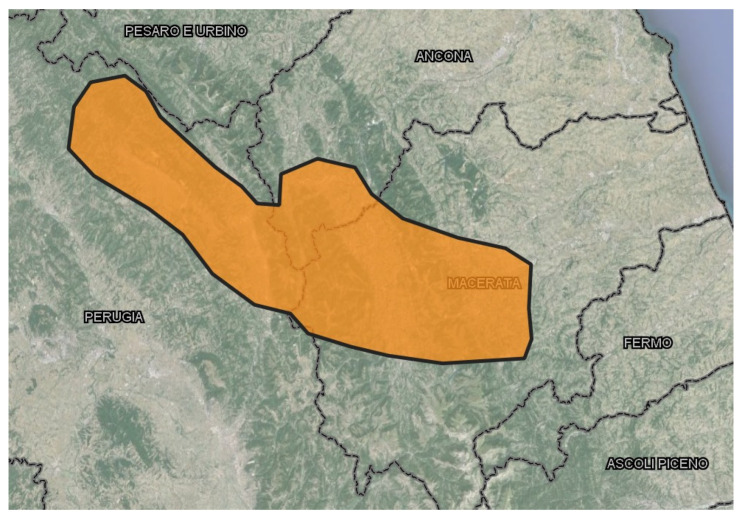
Sampling area in Umbria and Marche regions (central Italy).

The trial involved 280 wild boars collected from May 2018 to December 2023 in the Apennine area between the Umbria and Marche regions (central Italy) (Figure 1).

The numbers of animals sampled by year, gender, age, and weight after shooting are described in Table 1. Sampling of all the three matrices (swabs, liver, and feces) from each animal was not always possible.

Hunted animals were taken to the nearest registered collection center where biometric data (gender, age, and weight after shooting) were registered. Carcasses were eviscerated, refrigerated, and remained in the cold room (4 ± 1 °C) without skinning for up to 5 days. After the evisceration procedure, fecal samples were collected directly from the rectum and a portion of the liver was aseptically excised. The liver was considered as it represents an appreciated dish and is also an ingredient of local dry-fermented sausage production, by hunters and local food-processing plants [12]. Carcasses were transferred to the Game Handling Establishment (GHE), where they were skinned and inspected by a veterinary officer. Then, carcass surfaces were sampled with sterile moisturized sponges (3M Italia, Milan, Italy), which were rubbed over four areas of 100 cm^2^ each (rump, flank, brisket, and foreleg). All samples were kept under refrigerated conditions (4 °C) until microbial examination. In total, 258 and 226 samples of liver and carcass swabs, respectively, were collected from the 280 wild boars.

### 2.2. Microbiological Analysis

The detection of *Salmonella* spp. from feces was performed as described in ISO 6579-1:2020 [13]. Aliquots of 10 g of feces were placed into sterile Stomacher reinforced round bags (D.F.D s.r.l., Pavia, Italy) and 90 mL of buffered peptone water (Biolife Italiana s.r.l., Milan, Italy) was added. After incubation at 34–38 °C for 18 ± 2 h (pre-enrichment), 0.1 mL was inoculated into 10 mL of enrichment broth (Rappaport Vassiliadis soy broth, Biolife Italiana s.r.l., Milan, Italy), followed by incubation at 41.5 ± 1 °C for 24 ± 3 h. From this selective enrichment culture, 10 µL was spread onto two plates of selective and differential media, xylose lysine deoxycholate agar (Biolife Italiana s.r.l., Milan, Italy), and Chromogenic Salmonella Agar Base with *Salmonella* selective supplement (Biolife Italiana s.r.l, Milan, Italy) incubated at 34–38 °C for 24 ± 3 h.

Liver samples were removed from the transport bag and the liver surface was cauterized by a hot iron. Then, 25 g samples were aseptically collected from inside the liver by making a 1–2 cm deep incision in the cauterized area.

The 4 sponges per carcass were combined. *Salmonella* detection from sponges and liver samples (25 g) was carried out with an alternative technique based on an enzyme-linked fluorescent immunoassay, the VIDAS^®^ SLM test (bioMérieux, Marcy-l’Etoile, France), which has been validated by AFNOR (AFNOR BIO 12/16-09/05). Sponges and liver samples were pre-enriched in buffered peptone water (Biolife Italiana s.r.l., Milan, Italy), with incubation at 37 ± 1 °C for 16–22 h; thereafter, 0.1 mL was inoculated into 10 mL of SX2 enrichment broth (*Salmonella* Xpress; bioMérieux, Marcy-l’Etoile, France) and incubated at 41.5 ± 1 °C for 22–26 h. Broth cultures that tested positive in the immunoassay were confirmed, as previously described and outlined in ISO 6579-1:2020 [13].

Isolated strains were kept at 34–38 °C for 24 ± 3 h in nutrient agar (Biolife Italiana s.r.l., Milan, Italy) and then serotyped based on the White-Kauffmann-Le Minor scheme by the slide agglutination test [14,15].

Determination of aerobic colony counts (ACCs) and *Enterobacteriaceae* (ENT) of carcass samples was carried out following standard methods. In particular, ACCs were measured on the carcass samples according to ISO 4833-1:2022 [16]. After the initial suspension was prepared in buffered peptone water (Biolife Italiana s.r.l., Milan, Italy), the sample was diluted and included in plate count agar (PCA, Biolife Italiana s.r.l., Milan, Italy) and incubated at 30 ± 1 °C for 72 ± 3 h. ENT counts were performed in accordance with a validated alternative method of ISO 21582-2 [17] (AFNOR AES 10/07-01/08) on a chromogenic soil REBECCA^TM^ base (bioMérieux, Marcy-l’Etoile, France) with the addition of REBECCATM EB (bioMérieux, Marcy-l’Etoile, France) and incubated at 37 ± 1 °C for 24 ± 2 h. At the end of the incubation period, the colonies were counted and the colony-forming unit (CFU) number was calculated and transformed into log values.

### 2.3. Statistical Analyses

Prevalence analyses were performed by Epitools software (estimate true prevalence tool, Epitools, Ausvet, Fremantle, WA, Australia, https://epitools.ausvet.com.au/) [18]. Sensitivity and specificity were set at 0.9 and 0.99, respectively, and the confidence interval (CI) was set at 95%. For the evaluation of the difference between the average values of microbial loads in *Salmonella*-positive and -negative samples, an ANOVA test was carried out (Microsoft Excel, data analyses tools, Microsoft, Redmond, WA, USA) followed by Tukey’s test with significance set at *p* < 0.05. McNemar’s test was also performed on Excel data sheets to compare differences in the annual prevalence of *Salmonella* in carcass samples and it was considered significant with *p* < 0.05. The annual difference of Salmonella prevalence in the liver and fecal material was not analyzed because of the limited positivity detected.

### 2.4. Risk Assessment

In view of the prevalence results obtained on meat samples during the years of observation, a semiquantitative risk assessment was performed using the RISK RANGER software (https://foodsafetyportal.eu/riskranger/rr_riskranger.html (accessed on 5 March 2024)) [19,20,21]. The parameters considered are reported in Table 2. Two products were considered, fresh meat intended for cooking (meat preparations) and ready-to-eat short-time (15 days) dried fermented sausage, which is one of the most popular traditional game meat products in Italy [22,23] and can also contain liver [24]. The susceptibility and severity were set according to European Union Commission Notice 2022/C355/01 [25] that states that *Salmonella* must be considered as a severe hazard that could affect all members of a population. The probability of exposure to food was set considering the limited diffusion of the game meat consumption of the products (with a high frequency for dried fermented sausages, eaten mainly by hunters) and the population present in the two regions considered (2.2 million people) [26]. For the probability of food containing an infectious dose, the contamination of the raw products per serving was based on the prevalence observed in meat (carcass surfaces and liver). Fresh cuts are usually refrigerated under vacuum or frozen; therefore, no effect on microbial reduction was predicted [27]. In dry-fermented sausage the hurdle technology adopted usually eliminates the hazard [28]. Furthermore, in fresh cuts a recontamination is possible, while in fermented sausage this is more difficult due to the presence of the casing and the less favourable environment [29]. The post-processing control system was assumed to be well controlled under the implemented HACCP plan at GHEs and meat processing plants, with regular checks for *Salmonella* on carcass surfaces and meat products according to EC Regulation 2073/2005 [30]. This regulation also stipulates the absence of *Salmonella* in (5 × 25 g) the final products, highlighting that the presence of a single *Salmonella* cell is sufficient to pose a risk to consumers (to cause infection in consumers, there is no need for post-processing growth of *Salmonella*). The cooking of meat cuts is usually performed to eliminate the hazard while in a raw (fermented) ready-to-eat product, no effect of the preparation could be considered in a risk assessment. The results are given as the probability of illness per day, per consumer of interest; total predicted illnesses/annum in population of interest; and risk ranking from 0 to 100.

## 3. Results

During the hunting seasons considered, the number of animals positive for *Salmonella* was 8 out of 280 sampled wild boars (Table 3), with a prevalence of 2.86% (confidence interval, CI 95%, 1.45–5.54%). *Salmonella* spp. was detected in five carcass swabs, three liver samples, and one fecal sample. One subject was positive in both the liver and the feces, but with different *Salmonella* serotypes. Considering meat contamination, the prevalence was 2.21% (CI 95%, 0.95–5.07%) and 1.16% (CI 95%, 0.40–3.36%) in carcass swab and liver, respectively. Taking into account the year of observation, *Salmonella* prevalence in carcass swab was 5.17 (CI 95%, 1.77–14.14%), 1.85 (CI 95%, 0.33–9.77%), and 1.37 (CI 95%, 0.24–7.36%), in 2018, 2021, and 2023, respectively. In liver, *Salmonella* was detected only in 2022–2023 (prevalence 2.59%, CI 95%, 0.88–7.33%). *Salmonella Stanleyville* was isolated from six out of nine strains obtained from the positive samples and another three serotypes were also detected (*S.* Typhimurium, a monophasic variant of *Salmonella* Typhimurium 4,[5],12:i:-(MVST), and *S.* Derby). Positive animals were, on average, older than 24 months (average age of positive wild boars = 30.0 ± 6.41 months; negative animal average age = 27.73 ± 9.86 months) and had an average weight of 59.00 ± 9.11 Kg (average weight of negative wild boars = 60.23 ± 21.49 kg). Only one positive sample was detected in an animal with damage to the gut due to the shooting event, although 42 wild boars had damage to the gut due to shooting or improper evisceration.

The microbial counts performed on carcass swabs in positive and negative samples are reported in Table 4. No differences were detected between samples positive or negative for *Salmonella* for ACC or ENT counts.

For the risk-assessment analysis, conducted on the two considered products, the probability of illness per day, per consumer of interest was 2.30 × 10^−6^ and 9.21 × 10^−6^ for meat cuts and dry-fermented sausages, respectively. Meat cuts intended for cooking had a total predicted illnesses/annum in the population of interest of 1.01 × 10^2^ and fermented sausages had a total predicted illnesses/annum of 4.03 × 10^2^. The risk rank obtained was 60 for undercooked meat cuts and 64 for ready-to-eat dry-fermented (15 d) sausages. Only if meat preparations were properly cooked (meat preparation RELIABLY eliminates the hazards) was the risk score reduced to the minimum rank.

## 4. Discussion

*Salmonella* has been widely studied in wild boars through both indirect serological analyses and isolation from different organs and tissues [2,10]. Nonetheless, the updating of data on the prevalence and incidence of this pathogen in game meat and the serovars present in specific areas is crucial in order to comprehend the role that game animals play in spreading this zoonotic pathogen and how they can jeopardize control measurements implemented by veterinary authorities [31]. The tools used for this purpose are different and the results obtained could have different meanings. Serological analyses will only give information on the epidemiology of the disease in wild boar and the circulation of the microorganism in the environment [10,32,33], but do not provide information on the presence of viable *Salmonella*, and thus, on the real risk from wild boar meat consumption. Fecal sampling will provide further information on circulating serotypes and should be considered in sampling plans, as gut content could be a potential route for carcass contamination [26,28]. Sampling of lymph nodes will give information on the still-present *Salmonella* infection in the animal, with the risk that the pathogen is shed into the environment [34,35], but also on the possible spread of the microorganism during handling at the game-handling establishment (e.g., incision of tonsils or mandibular/ileocecal lymph nodes during evisceration could pose a risk of carcass contamination) [36]. In these samples, a high prevalence of *Salmonella* is generally detected, and this is likely to lead to overestimation of the real prevalence in the meat [33,37,38,39].

In the present survey, the prevalence was evaluated by direct sampling of carcass surfaces and the liver, which could be considered meat, and therefore the results could be reliably used in risk-assessment models. Feces were sampled too, but with the aim of identifying a possible relationship to carcass or liver positive samples. The prevalence detected on the carcass was 2.21% with no substantial variation during the years of observation. Several authors have reported the absence of *Salmonella* spp. on carcass surfaces [40,41,42,43,44] and meat [45,46,47], whereas other authors have reported a prevalence similar to those found in our survey, in nearby geographic areas of Italy (0.9% in carcass swabs from the Campania region; 1.1% in carcass swabs from the Emilia Romagna region [39]; from 2.0% to 6.0% in carcass swabs from the Emilia Romagna region [48]; 3.9% in muscles from the Lazio region [49]; and 4.55% in meat cuts from the Tuscany region [50]) or in other countries (1.4% in carcass swabs and 1.9% in meat samples from Serbia [51]; 1.2% in carcass swabs from Spain [52]; and 3.1% in carcass samples from Serbia [53]. Higher prevalence values have been reported for *Salmonella* spp. in the Campania region (South Italy), with 7 muscle samples positive out of 22 (31.8%) [54], and in a specific hunting ground in the Vojvodina region of Serbia (33.3%) [53]. When a high prevalence is detected, specific evaluations on the diffusion of the pathogens in the area, as well as on the hygienic practices adopted during carcass dressing, are needed [51].

Indeed, *Salmonella* contamination refers mainly to fecal contamination of the carcasses due to gut rupture either in the harvest phase (i.e., shot position) or improper evisceration or contamination during skinning [42]. The detection of *Salmonella* spp. on carcass surfaces of only 1 subject out of 42 animals (shot in the abdomen or with rupture of the gut due to evisceration) and the limited percentage of positive fecal samples, means that there is a need for further investigation of the dynamics of carcass contamination. Other authors have detected *Salmonella* in the same number of carcasses shot in the head or in the heart compared to animals that had been shot in the abdomen [48]. The possible presence of *Salmonella* spp. in lymph nodes but not in the feces, the evisceration procedure performed (time and method), and the hygiene practices during handling should be taken into consideration for a deeper understanding of this dynamic. Furthermore, in our survey, carcass sampling was performed in GHEs registered according to EC Regulation 853/2004, with specific training of the operators to guarantee that hygiene practices are performed during operations. When proper training of the operators is performed, the hygiene level of the carcass, and therefore the possible contamination by *Salmonella,* can be minimized [51]. Mirceta et al. [51] also noted that higher microbial loads were found on carcass surfaces found positive for *Salmonella* spp., but in our study no difference was detected for ACCs or ENT between carcasses positive and negative for the pathogen.

Regarding the liver, the presence of *Salmonella* in this organ could be due to contamination from the gut, but also to its presence inside the organ in live animals without any other sign of the disease. In order to exclude possible external contamination, only the deep part of the organ was sampled, and no lesions were, indeed, observed on the sampled liver. The prevalence registered in our survey could be considered low when compared with those reported by other authors [55,56]. Nonetheless, some authors have reported the absence of *Salmonella* in wild boar livers [47]. The presence of *Salmonella* in the liver must be taken into consideration in risk assessment as this organ is regularly consumed by hunters and could enter in the food chain from both fresh use and fermented meat product manufacturing [12,57].

Some authors have studied the relationship between positivity for *Salmonella* and biometrical characteristics of the animals, but this was based on sampling of lymph nodes and feces [32]. The authors report a higher prevalence in animals under 20 kg (60% of the total), older than 24 months of age (43.4% of the total), and female (56.6% of the total). In our survey, *Salmonella* was isolated from wild boar carcasses and the liver of animals that were all over 24 months of age and in the weight range of 46 to 73 kg; however, no difference was found between male and female animals. Further studies are needed to determine if specific animal classes could be investigated for a higher probability of *Salmonella* spp. in their meat. Indeed, cross contamination could also occur when other animals, especially pigs, are slaughtered in the same GHE, as they are reported to have a higher prevalence of *Salmonella* spp. on carcass surfaces than wild boars [58,59]. This should be considered in epidemiological studies performed by sampling carcass swabs or meat cuts. Serotype analyses, as well as whole-gene sequencing, should therefore be considered, especially if outbreaks occur [39].

The main serotype detected in our study was *S. Stanleyville*, which has been reported as one of the most common serovars in wild boar by other authors (in meat [54] and mesenteric lymph nodes and feces [32]). The other serotypes detected have been reported in literature in wild boars, albeit with a different prevalence. Prevalence of *S. Typhimurium* ranged from 1.8% of isolates from fecal samples [33] to 29% of the isolates from lymph nodes, feces, and carcass sponges from wild boars [39]. A particular discussion should be made for *S. Derby* detected on carcasses and MVST detected in a fecal sample. *S. Derby* is reported as one of the top five EU-level *Salmonella* serovars involved in human salmonellosis [6] with pigs as main sources, and only one author has reported this serovar in wild boar [58]. Indeed, the hypothesis of a cross contamination with pigs should be considered in our case as the GHE where sampling was performed is intended for pig slaughtering too, albeit at a different time and after a proper cleaning and disinfection procedure of the structure and the equipment has been performed. MVST too, is frequently isolated from pigs [6], and in our survey, it was also isolated from wild boar feces, which excludes a bias due to cross contamination. The possible contact between wild boars and pigs could therefore be suggested even if pig farms in the area adopt proper biosecurity protocols and no food sources are shared between wild boars and pigs. Nonetheless, when comparing the serotypes and PFGE types of isolates from wild boars with those of farmed pigs in the same area, some authors report very little or no overlap, which suggests that the two animal populations are significantly separated in terms of infectious contacts [32].

The results of the semiquantitative risk assessment performed reveal that, when *Salmonella* prevalence is low and game meat is not eaten by all the people in the region, the possibility of illness in the population considered is low (rate of 4.6 per 100,000 people per year and 19 per 100,000 people per year for fresh meat cuts and dry sausages, respectively), but not absent. The intervention strategy to reduce the risk could be directed at the harvesting and handling of the animals (prevention), but a deeper analysis of meat contamination dynamics is needed. If preventative measures are not effective, the preparation method adopted for meat cuts could be the key to minimizing the risk [60]. This is mainly due to different habits in processing and preparing foods, as cooking is a crucial step in eliminating foodborne pathogens [61]. Traditionally, the preparation of game meat is performed by marinating it for several hours and performing a prolonged cooking [62] reducing the *Salmonella* risk. Nonetheless, some products could be prepared with new methods and techniques (low temperature and a long cooking time [63]). New consumer demands such as for minimally processed meat (e.g., carpaccio [64]) are also emerging. Therefore, to reduce the risk, a possible strategy should be the decontamination of meat cuts [65] or a campaign of risk communication to consumers and inserting the “need for proper cooking” on wild boar meat labels. Nonetheless, the cooking process surely minimizes the risk of contracting disease but it is not applied to all game meat products, such as dried, salted, cured or fermented meat products. In this case, a proper hurdle technology is needed and should be applied, as described for pork [66]. The food business operator, even with the aid of challenge tests, should demonstrate to the competent authority the effectiveness of the process to eliminate Salmonella from ready-to-eat fermented sausages obtained from wild boar meat.

## 5. Conclusions

This research highlights that the prevalence of *Salmonella* detected in other Italian and international surveys is consistent with the prevalence found in wild boars in the two Italian regions considered. However, the situation has to be constantly monitored to provide a time–geographical map of the infection in wildlife and possible control and prevention strategies for food safety. Moreover, *Salmonella* monitoring could be performed at GHE in accordance with EC Regulation 2073/2005 [30] for pigs, also setting process-hygiene or food-safety criteria for *Salmonella* in wild boar carcasses. Indeed, the EC Regulation does not consider process-hygiene criteria for game carcasses as it should, and as other authors have already pointed out [11].

In our study, the characterization of the involvement of particular serotypes in wild boar contamination and the definition of their role in meat safety were not possible due to the small number of *Salmonella*-positive samples, but they are crucial especially during food poisoning outbreaks. Moreover, further studies on an increased number of samples are also needed to define the main dynamics of *Salmonella* carcass contamination, according to the harvest methods and the GHE processing procedures, and the importance of the serotypes isolated. Our preliminary risk assessment, which used a semi-quantitative methodology, suggested that the risk was low but not zero, although a more detailed risk assessment should be performed. Therefore, control strategies to reduce or eliminate *Salmonella* in the game meat chain are needed for wild boar meat and meat products.

## Figures and Tables

**Table 1:** Number of wild boars sampled in relation to year, gender, age, and weight after shooting.

		N° Wild Boars	Carcass	Liver	Feces
Year	2018	58	58	58	32
	2019	31	31	28	21
	2021	54	51	52	38
	2022	10	10	10	9
	2023	127	76	110	74
Gender	Male Female	126 154	101 115	119 139	85 89
Age (months)	12 24	46 117	35 97	41 108	22 85
	36	100	79	92	52
	48	17	15	17	15
Weight after shooting (kg)	0–30 31–60	23 142	20 116	23 128	15 88
	61–90	91	72	85	56
	>90	24	18	22	15

**Table 2:** Parameters considered in the risk assessment for *Salmonella* in wild boar meat.

Risk Ranger	Fresh Game-Meat Cuts	Dry-Fermented Sausages
Hazard severity	SEVERE hazard—causes death to most victims	SEVERE hazard—causes death to most victims
How susceptible is the population of interest?	GENERAL—all member of the population	GENERAL—all member of the population
Frequency of consumption	A few times per year	Monthly
Proportion of consuming population	Some 25%	Some 25%
Size of consuming population	2.2 million people	2.2 million people
Probability of contamination of raw product per serving	Prevalence detected in carcass surfaces and liver	Prevalence detected in carcass surfaces and liver
Effect of processing	The process has NO effects on the hazards	The process USALLY (99%) eliminates the hazards
Is there potential for recontamination after processing?	Yes, minor (1% frequency)	No
How effective is the post-processing control system?	Well controlled	Well controlled
What increase in the post-processing contamination level would cause infection or intoxication to the average consumer?	None	None
Effect of preparation before eating	Meat preparation USUALLY eliminates (99%) hazards	Meat preparation has NO effect on the hazards

**Table 3:** Positive samples to *Salmonella* spp. in wild boar in the years 2018–2023.

Year	Gender	Age (Months)	Weight (Kg)	Shoot Localization	Positive Sample	Serotype
2018	Male *	24	51	Neck	Carcass swab	*S.* Typhimurium
2018	Male *	24	60	Thorax	Carcass swab	*S.* Stanleyville
2018	Male **	36	73	Head	Carcass swab	*S.* Stanleyville
2021	Male ***	36	65	Multiple (including abdomen)	Carcass swab	*S.* Stanleyville
2022	Female *	24	55	Thorax	Liver	*S.* Stanleyville
					Fecal	MVST
2023	Male **	36	46	Head	Liver	*S.* Stanleyville
2023	Female **	36	63	Head	Liver	*S.* Stanleyville
2023	Female *	24	60	Head	Carcass swab	*S.* Derby

The positive samples in the same year were obtained from animals collected on different days. * = Carcass swabs and liver and fecal material were collected from the same wild boar; ** = fecal material was not collected; *** = fecal material and liver were not collected.

**Table 4:** Microbial counts in Salmonella-positive and -negative carcasses. Average values in Log CFU/400 cm^2^ (±standard deviation).

	*Salmonella*-Positive	*Salmonella*-Negative	*p* Value
ACC	4.59 (±0.84)	4.66 (±1.71)	0.94
ENT	2.89 (±0.75)	2.73 (±1.67)	0.84

ACC = aerobic colony count; ENT = *Enterobacteriaceae* count.

## Data Availability

The original contributions presented in the study are included in the article, further inquiries can be directed to the corresponding author.
